# Fellow-Workers and the Roll of Honour

**Published:** 1914-10-17

**Authors:** 


### ROUND THE WORLD.

[The Editor Invites Early Information and Contributions Marked "Fellow-Workers."]

### Fellow-Workers and the Roll of Honour.

Sir William Milligan, who holds the rank of major in the 2nd Western General Hospital of the Territorial Establishment at Manchester, is one of the leading authorities on diseases of the ear, nose, and throat in this country. An M.D. of Aberdeen with highest honours, Sir William Milligan has been for many years associated with Manchester, where he is honorary surgeon to the Ear Hospital, lecturer on diseases of the ear in the University, and consulting aural surgeon to the Warehousemen and Clerks' Association.

The captains of the 2nd Western General Hospital, in which, it should be noted, no fewer than seven members of the staff are University professors, are: Mr. J. P. Buckley, Dr. W. J. S. Bythell, Dr. E. N. Cunliffe, Dr. D. E. Cope, Dr. A. Donald, Dr. W. H. P. Hey, Mr. W. A. Hooton, Dr. G. E. Loveday, Dr. R. W. Marsden, Dr. F. C. Moore, Dr. A. Sellers, Mr. E. D. Telford, Dr. F. E. Tylecote, Mr. S. R. Wilson, Mr. A. Wilson, Dr. R. T. Williamson, Mr. P. R. Wrigley, Dr. J. Wharton, Dr. C. H. Melland, Dr. G. Wright, and Mr. H. H. Rayner.

The majors at the 3rd Western General Hospital, Cardiff, are Dr. B. W. Broad, Mr. W. F. Brook, Dr. D. R. Griffiths, Dr. A. Howell, Dr. C. Lewis, and Dr. D. R. Paterson. The captains in the same establishment being: Mr. R. C. Elsworth, Mr. C. A. Griffiths, Mr. W. J. Greer, Mr. W. Nicholson, Mr. E. Reid, Dr. H. A. Schilberg, Mr. F. G. Thomas, and Mr. T. M. Thomas.

Mr. William Frederick Brook, F.R.C.S., practises at Swansea, where for some years he has been on the staff of the General Hospital. He is an old St. Thomas's man, and as a junior practitioner held the posts of house-surgeon at the Great Ormond Street Children's Hospital and at the West London Hospital, Hammersmith. Mr. Brook is a medical referee under the Workmen's Compensation Act, and was formerly surgeon to the Throat and Ear Department of the Swansea Hospital. He is also an ex-president of the South Wales branch of the British Medical Association.

Dr. Benjamin Wm. Broad, M.B., C.M. Edin., is the resident medical superintendent of Cardiff Sanatorium. He is particularly interested in all public health matters, and is an associate of the Society of Medical Officers of Health. Formerly Dr. Broad was house-physician to the City and Canongate Hospital at Edinburgh.

Dr. Alfred Howell, M.D. Lond., is assistant physician to King Edward VII.'s Hospital at Cardiff. Entering St. Mary's Hospital Medical School in 1892 with the senior entrance scholarship in natural science, he subsequently qualified M.R.C.S., L.R.C.P., and held the posts of resident obstetric officer and demonstrator of pathology there. Dr. Howell also won two general proficiency scholarships as a student, and before settling in Wales was for a time assistant medical officer to the Tooting Fever Hospital.

Dr. Cyril Lewis, M.D. Edin., holds important appointments at Cardiff, including that of physician to King Edward VII.'s Hospital, where he was formerly anæsthetist. He is also anæsthetist to the Royal *Hamadryad* Seamen's Hospital, and honorary medical referee to the Royal National Consumption Hospital at Ventnor, an institution to which he was in earlier years attached as senior resident medical officer. Among Dr. Lewis's other past appointments may be mentioned those of house-physician to the Metropolitan Hospital and assistant medical officer to the Joint Counties Asylum at Carmarthen.

Mr. Donald Rose Paterson, M.D. Edin., M.R.C.P. Lond., is consulting surgeon to the ear, nose, and throat department of the Royal *Hamadryad* Seamen's Hospital, and surgeon to the ear, nose, and throat department of King Edward VII.'s Hospital at Cardiff. The author of various contributions on laryngology and otology, as well as the translator of Professor Killian's "Accessory Sinuses of the Nose," Mr. Paterson studied his special subject in Vienna and Berlin, as well as at Edinburgh, where he spent his student years.

Mr. Philip Rhys Griffiths, M.B., B.S. Lond., M.R.C.S. Eng., qualified as a student of University College Hospital in 1880. He is well known in Cardiff, where he has taken considerable interest in public work, and after many years' service he is now senior surgeon to King Edward VII.'s Hospital. Formerly president of the Cardiff Medical Society, in earlier years Mr. Griffiths was lecturer in physiology and hygiene in the technical school attached to University College, Cardiff. He was also at one time clinical assistant to the Royal Ophthalmic Hospital, the Central London Throat and Ear Hospital, and to the Hospital for Women in Soho Square.

The last-named must not be confused with Mr. Cornelius Albert Griffiths, who holds the rank of captain in the same Territorial hospital, and is also a member of the surgical staff of King Edward VII.'s Hospital. Mr. C. A. Griffiths, who is a Bristol and Bart.'s man, qualified in 1889, and became an F.R.C.S. Eng. in 1893. After qualification he held several important appointments in Bristol, including those of medical tutor, assistant house-surgeon, and assistant physician to the General Hospital.

Dr. Mitchell William Stephens, also of Cardiff, possesses a fine academic record. A University College student, he qualified by taking the conjoint diploma in 1891, and in the same year became an M.B. Lond., with a University scholarship and gold medal in medicine and first-class honours in obstetric and forensic medicine. In the following year he was successful in the M.D. Lond. degree, in which he qualified for a gold medal. Proceeding from Cardiff, he followed up his successes there by becoming a member of the staff of the big local hospital, as well as of University College, Cardiff. Dr. Stephens' present appointments include those of lecturer in pharmacology at the last-mentioned institution, senior physician to King Edward VII.'s Hospital, examiner in pharmacology for the University of Wales, as well as chairman of the Cardiff division of the British Medical Association.

